# NLRX1 emerges as the only observed essential regulator of the mPTP, challenging mPTP inhibition as a valid target of cardioprotection

**DOI:** 10.1007/s00395-026-01204-6

**Published:** 2026-08-19

**Authors:** Kielen R. Zuurbier, Coert J. Zuurbier

**Affiliations:** 1 Department of Molecular and Cell Biology, University of California, Berkeley, CA USA; 2 Howard Hughes Medical Institute, University of California, Berkeley, CA USA; 3 L.E.I.C.A., Department of Anesthesiology, Amsterdam UMC, Cardiovascular Sciences, University of Amsterdam, Amsterdam, The Netherlands

**Keywords:** NLRX1, mPTP, Mitochondria, Innate immunity, Ischemia-reperfusion injury, Cardioprotection

## Abstract

The mitochondrial permeability transition pore (mPTP) opening is a phenomenon in which the inner mitochondrial membrane abruptly becomes permeable when matrix calcium reaches a critical threshold. Despite 5 decades of intensive research, no protein has been universally accepted as essential for mPTP opening, limiting mechanistic understanding and raising questions about the validity of mPTP-targeted strategies to mitigate cardiac ischemia–reperfusion (I/R) injury. Here, we discuss convergent findings from two independent laboratories identifying the innate immune receptor NLRX1 as an unexpected, essential requirement for mPTP activity. NLRX1 is the only NOD-like receptor (NLR) that is targeted to the mitochondrion. NLRX1 deficiency abolishes (1) calcium-induced mPTP opening, (2) cyclosporine A sensitivity of the pore, and (3) mitochondrial calcium release following cardiac I/R. To test whether loss of mPTP function aligns with loss of NLRX1 across evolution, we performed forward and reciprocal bioinformatic (Blastp) searches and found that species reported to lack an mPTP (e.g., *Artemia franciscana* and *Drosophila melanogaster*) also lack NLRX1, further supporting a mandatory role for NLRX1 in mPTP occurrence. Notably, NLRX1-deficient hearts can exhibit increased, rather than decreased, I/R injury at specific ischemia durations. This mirrors reports that deletion of established mPTP regulators (e.g., *Ppif*) may also worsen injury under defined conditions, consistent with context-dependent, potentially protective roles for transient mPTP activity (e.g., mitochondrial calcium release, PI3K/Akt signaling). In summary, we propose that NLRX1 is the only currently identified protein that is strictly required for mPTP opening, and that indiscriminate inhibition of the mPTP is unlikely to represent a universally effective cardioprotective strategy against I/R injury.

## The mPTP, its undefined components, and suggested role in cardiac I/R and protection

The mitochondrial permeability transition pore (mPTP) refers to a sudden increase in permeability of the inner mitochondrial membrane to molecules up to 1.5 kDa in size via an as‐yet undefined pore that forms when matrix calcium reaches a critical threshold. This pore opening was first described more than 70 years ago [24, 30, 50]. The mPTP has since been implicated in numerous pathological conditions, including ischemia–reperfusion injury (IRI), aging, Parkinson’s disease, Alzheimer’s disease, and Duchenne muscular dystrophy in mice [4, 21, 36, 43, 47]. Human data are still largely missing.

The involvement of the mPTP in cardiac ischemia–reperfusion injury appears logical, given that ischemia and early reperfusion are characterized by rapid rises in intracellular calcium to pathological high levels [3, 56].

For the past two decades, the prevailing paradigm in cardioprotection against ischemia–reperfusion injury (IRI) has been that mitochondria serve as the final common end-effector of cell death with prevention of mitochondrial permeability transition pore (mPTP) opening as central therapeutic target [23, 26]. This concept was supported by a large body of preclinical experimental evidence demonstrating that inhibition of the mPTP reduces myocardial infarct size in multiple models of cardiac I/R. Following the seminal observation that cyclosporine A (CsA), via inhibition of cyclophilin D (CypD), protects the heart from I/R injury [20], numerous studies confirmed cardioprotective effects of mPTP inhibition in experimental settings.

However, inconsistencies soon emerged, indicating that inhibition of the mPTP—particularly through targeting CypD—does not universally confer protection and may, under certain conditions, also exacerbate injury. For example, Ruiz-Meana and co-workers [52] demonstrated that deletion of CypD increased infarct size following 30 min of global ischemia in isolated mouse hearts, whereas infarct size was reduced only when ischemia duration was extended to 60 min. One plausible explanation for this paradox is that, after relatively short ischemic insults, cardiomyocyte death is predominantly mediated by sarcolemmal rupture rather than by mPTP-dependent mitochondrial dysfunction. Under these conditions, intact mitochondria rapidly regenerate ATP upon reperfusion, thereby fueling excessive calcium-activated hypercontracture that mechanically disrupts the plasma membrane [18].

Similarly, in an in vivo model of cardiac I/R, Ikeda and co-workers [31] reported that CypD deletion conferred protection after intermediate ischemia durations (30–45 min) but failed to reduce infarct size following more prolonged ischemia (60–90 min). Notably, infarct size after extended ischemia was still attenuated by deletion of the C–C chemokine receptor 2 (CCR2), highlighting that at longer ischemic durations inflammatory mechanisms become dominant contributors to injury and can still be therapeutically targeted independently of the mPTP.

In addition, accumulating evidence indicates that mPTP opening is not always deleterious. Transient mPTP opening likely contributes to the maintenance of mitochondrial homeostasis, possibly by functioning as an important mitochondrial calcium release pathway [49].

Despite this dual role, prevention of mPTP opening has become a central objective in cardioprotection research and has been regarded as a near “holy grail” strategy over the past 2–3 decades [21, 27]. In clinical studies with PCI in acute myocardial infarction patients, the involvement of mPTP in reperfusion injury was studied through the application of cyclosporine A (CsA) with neutral and disappointing results [14, 48]. CsA is known to be a potent inhibitor of the Ca^2+^-induced pore in cardiac mitochondria through binding to cyclophilin D as regulator of the mPTP and most preclinical studies have shown cardioprotection with CsA [13, 22]. However, CsA is not specific for pore inhibition, because the binding of CsA to CyPD also results in inactivation of calcineurin and consequently reduced T-cell activation. In addition, further research also demonstrated that CsA not consistently impaired pore opening, with efficacy being dependent, among other conditions, on metabolic substrates and aging [15, 42]. Thus, although these first clinical studies directed at mPTP modulation show no support for mPTP being a valuable target for human cardioprotective intervention, it cannot be excluded that the neutral effects of CsA treatment were due to none-specificity and off-targets effects, such that clinical studies with specific inhibition of the mPTP are still awaited.

Various proteins have been proposed as essential structural components of the mitochondrial permeability transition pore, including the voltage-dependent anion channel (VDAC) [11, 66], adenine nucleotide translocase (ANT) [8], mitochondrial phosphate carrier (PiC) [37], cyclophilin D (PPIF) [6, 46], and mitochondrial F_1_F_o_-ATP synthase in the form of dimers, monomers, or the c-subunit ring alone [2, 19]. However, the persistence of mPTP activity under high calcium conditions in which these proteins were genetically ablated, together with the maintained sensitivity of the pore to cyclosporine A (CsA) in most of these knockout models (except for CypD KO), has argued against an essential role for any of these components in mPTP formation. Consequently, these proteins are now generally regarded as important regulators of the mPTP rather than indispensable structural constituents [7, 10, 25, 32, 33, 35], and the field is still waiting for proteins that are essential for the mPTP opening to occur.

Recently, ATAD3 was reported in a preprint as a newly discovered essential mPTP pore candidate [51]. Indeed, genetic ablation of the Atad3 gene in mouse resulted in much increased Ca 2 + retention capacity, with ATAD3 protein having intrinsic channel activity. In addition, cardiac-specific ATAD3 deletion reduced infarct size after 60 min of ischemia without additive protection by CsA. However, ATAD3 did not show up as a putative component of the mPTP in the unbiased search for essential mPTP components in human cells [60]. One possible reason why ATAD3 was not detected in the unbiased screen in human cells is that there are three ATAD3 paralogs in human cells, whereas only one in the mouse. Further research in human-based cells and tissue models are needed to examine whether ATAD3 also function as essential mPTP component in humans.

## NLRX1, the first essential protein of the mPTP

Recently, two independent studies have identified the innate immune receptor NLRX1 as a novel and potentially essential component of the mPTP. In a murine study from our laboratory investigating the role of NLRX1 in ex vivo cardiac ischemia–reperfusion injury, ablation of NLRX1 was shown to result in: (1) complete loss of calcium-induced, sudden pore opening in isolated cardiac mitochondria; (2) loss of CsA-mediated delay of pore opening; and (3) increased mitochondrial calcium accumulation following cardiac ischemia–reperfusion interventions [62]. Subfractionation experiments localized NLRX1 to the inner mitochondrial membrane of cardiac mitochondria, consistent with the proposed location of the mPTP. Together, these findings provide strong evidence that NLRX1 is essential for mPTP formation and function.

In a subsequent study from the laboratory of David Clapham, a genome-wide CRISPR–Cas9 fluorescence-activated cell sorting (FACS) screen was performed to identify genes conferring resistance to mitochondrial calcium overload in human haploid HAP1 cells [60]. This screen assessed 19,112 genes using a receptor–ligand fluorescent assay based on a mitochondrial HaloTag receptor (HaloMTS) and an Oregon Green-conjugated HaloTag ligand to monitor mitochondrial calcium dynamics. Among all genes tested, *NLRX1*, *Ppif*, and the nuclear chromatin-binding protein *BPTF* emerged as the top three hits conferring the greatest resistance to calcium overload.

To validate these findings, individual knockout cell lines for each candidate gene were generated and subjected to a calcium retention capacity (CRC) assay. In such assay, isolated mitochondria are subjected to separate pulses of Ca2 + with fluorescence monitoring of calcium outside of the mitochondria. Each pulse of calcium is quickly followed by the disappearance of calcium in the medium, because it is taken up by the mitochondria. However, when a certain threshold of calcium is reached (capacity), the mitochondrial pore is opened and calcium rushes out of mitochondria resulting in a sharp increase in fluorescence. This calcium threshold is called the CRC. CRC was markedly increased in *NLRX1*-deficient cells compared with *Ppif*- and *BPTF*-deficient cells. Notably, calcium-induced mitochondrial calcium release was absent in both *NLRX1* and *BPTF* knockout cells but remained detectable in *Ppif* knockout cells. Furthermore, cyclosporine A (CsA) failed to exert any effect in *NLRX1*-deficient cells, while CsA responsiveness was preserved in *BPTF* knockout cells [52].

Based on these observations, the authors concluded [60] that only ablation of the mitochondrial matrix protein NLRX1 simultaneously increased CRC, abolished calcium release, and rendered the response CsA-insensitive. As such, NLRX1 was identified as the sole essential activator of the human, CsA-sensitive mPTP in this near-genome-wide screen. To date, NLRX1, therefore, represents the only protein demonstrated to be strictly required for mPTP occurrence.

## NLRX1 in pathology and health

Nucleotide-binding oligomerization domain (NOD)-like receptors (NLRs) are intracellular pattern-recognition receptors that sense microbial- and danger-associated molecular patterns and form an integral part of the innate immune system [68]. Among this family, NLRX1 is unique in that it localizes to mitochondria and is generally regarded as an anti-inflammatory regulator of cellular stress responses [5]. NLRX1 shows highest expression in the heart, skeletal muscle, and mammary gland, and has been implicated in the regulation of mitochondrial reactive oxygen species (ROS) production, as well as calcium-dependent sensitivity to apoptosis initiation [1, 54, 58]. Mitochondrial ROS production figures prominently in the regulation of the mPTP.

In the heart, NLRX1 expression is reduced following ischemia–reperfusion (I/R) injury in multiple species, including mouse, pig, and human hearts [38, 62]. Importantly, this post-ischemic downregulation of NLRX1 may be driven, at least in part, by increased expression of microRNA-15 following ischemia, as miR-15 has been shown to negatively regulate NLRX1 expression [29, 59].

Deletion or deficiency of NLRX1 has been consistently associated with exacerbation of tissue injury across multiple organ systems. In experimental models, loss of NLRX1 aggravates ischemia–reperfusion injury in cardiac cells, epithelial cells, kidneys, and whole hearts [38, 57, 62, 63]. Vice versa, NLRX1 overexpression resulted in protection against ischemia–reperfusion injury for cardiac cells [38], kidney cells [40], and intestine [39]. Furthermore, NLRX1 deficiency increases endoplasmic reticulum stress and promotes cardiac hypertrophy [45], worsens pulmonary hypertension and inflammatory responses [61], and impairs mitochondrial homeostasis and quality control, leading to increased cellular senescence [55].

Consistent with a role in cardiovascular health, cardiac NLRX1 mRNA levels decline during the development of heart failure [12], and NLRX1 expression also decreases with aging in lung tissue [53]. Collectively, these findings position NLRX1 as a key regulator of mitochondrial function, stress resilience, and tissue integrity, highlighting its broad relevance across aging- and injury-related pathologies.

NLRX1 plays an important regulatory role in cellular and organ metabolism across multiple tissues, including the heart and immune cell populations.

**Fig. 1:**
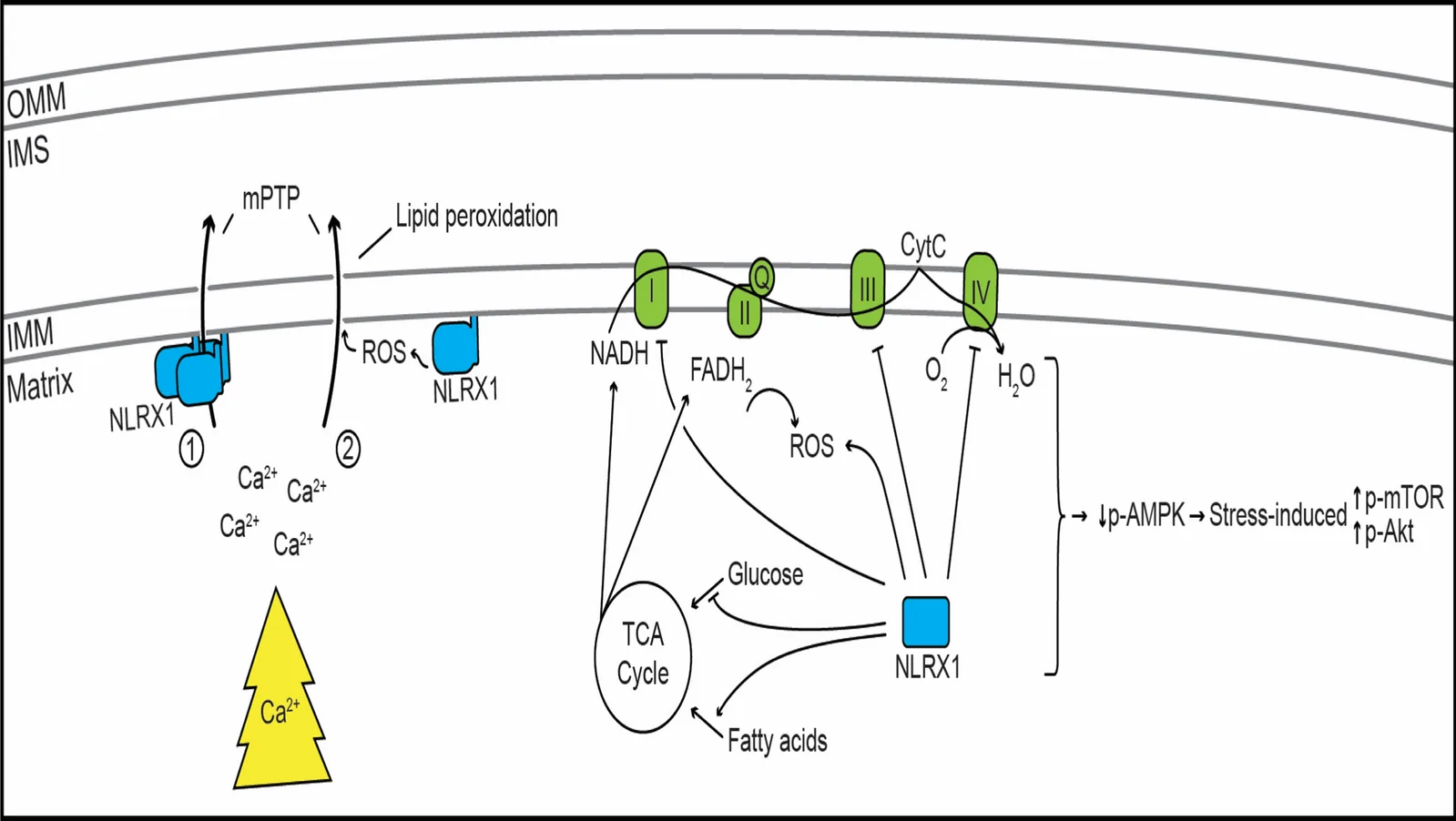
NLRX1 effects on mPTP, electron transport chain (ETC), oxygen consumption, metabolism, and stress signalling in the heart. Calcium-induced mPTP opening is either facilitated through NLRX1 directly forming the pore (1) or through NLRX1-mediated ROS causing the formation of peroxidized lipids that form the pore (2). NLRX1 inhibits the ETC resulting in ROS production and impaired oxygen consumption. The presence of NLRX1 maintains AMPK in a low-activated state, allowing unimpaired stress activation of the RISK pathway and mTOR

In general, mitochondrial localization of NLRX1 imposes a restraining influence on mitochondrial metabolism and oxidative phosphorylation, as evidenced in hearts, macrophages, and epithelial cells [41, 57, 63]. In macrophages, genetic depletion of NLRX1 results in increased basal and maximal oxygen consumption rates and enhanced respiratory capacity, accompanied by a functional polarization toward a profibrotic M2 phenotype [41]. Similarly, isolated hearts from NLRX1-deficient mice exhibit elevated oxygen consumption together with a pronounced metabolic shift characterized by increased glycolysis and glucose oxidation at the expense of fatty acid oxidation [63]. Similarly, in permeabilized cardiac fibers, ablation of NLRX1 resulted in slowed mitochondrial responses to fatty acids, and an activation of the energy sensor AMPK in isolated hearts, indicative of metabolic stress in NLRX1-deficient hearts (Fig. 1) [62].

More recently, mitochondrial NLRX1 has been identified as a metabolic sensor that acts as an essential regulator of mitophagy independently of the canonical PINK–Parkin pathway [64]. In this context, NLRX1 functions as a metabolic checkpoint by monitoring and binding cytosolic acetyl-coenzyme A (AcCoA) levels. Under conditions of reduced AcCoA availability, the autoinhibited state of NLRX1 is relieved, promoting NLRX1 oligomerization and subsequent recruitment of microtubule-associated protein 1 light chain 3 (LC3), thereby initiating the mitophagy cascade.

Collectively, these findings establish mitochondrial NLRX1 as a multifunctional and versatile protein that integrates key cellular signals—including calcium, reactive oxygen species, and intermediary metabolites—to modulate mitochondrial metabolism, quality control, and overall mitochondrial health (see Table 1 and Fig. 1).

**Table 1:** NLRX1 Effects on the heart

Effects	Experimental details	(Patho)Physiological implications	Reference
↑mPTP	Isolated mouse heart; human HAP1 cell	Enable post-I/R mito calcium release	[60, 62]
↑mitophagy	Various cell types; mouse liver	Facilitate metabolically regulated (low AcetylCoA) mitophagy*	[64]
↓MVO_2_	Isolated mouse heart	Reduced energy demand of the heart	[63]
↓p-AMPK	Isolated mouse heart	Facilitating post-I/R activation of PI3K/Akt protective signalling	[62, 63]
↑FA/CA metabolism	Isolated mouse heart; permeabilised cardiac fibres	Increased FA over CA metabolism, may reduce heart failure	[62, 63]
↓ER stress	In vivo mouse heart; H9C2 cells	Decreased cardiac hypertrophy and inflammation	[45]
↓sepsis	In vivo mouse heart; primary mouse cardiac cells	Protection against sepsis with reductions in inflammation	[65]

Cardiac effects of NLRX1 present in heart versus NLRX1-deficient heart. *mPTP* mitochondrial permeability transition pore; *MVO*_*2*_ oxygen consumption of the heart in (μmol/min/G dry weight); *p-AMPK* phosphorylated AMP-activated protein kinase; *FA/CA metabolism* ratio of fatty acid metabolism over carbohydrate metabolism; *ER* endoplasmic reticulum

*This NLRX1 effect has not been proved in hearts yet

## NLRX1 and mPTP across species

An additional strategy to identify essential components or regulators of the mitochondrial permeability transition pore is to examine species reported to possess, or lack, a functional mPTP [17]. A well-characterized example of a species in which the mPTP is absent is the anoxia- and salt-tolerant brine shrimp *Artemia franciscana* [44]. Notably, *A. franciscana* mitochondria express several proteins previously proposed to regulate or constitute the mPTP, including adenine nucleotide translocase (ANT), cyclophilin D (CypD), and the F_1_F_o_-ATP synthase. The persistence of these components despite the apparent absence of mPTP activity suggests that loss of the pore cannot be explained by the absence of any of these frequently proposed regulators alone [44].

Similarly, *Drosophila melanogaster* has been reported to lack a characteristic calcium-induced mPTP [9], and although mPTP-like mechanisms were proposed for *Caenorhabditis elegans*, the mPTP has never been demonstrated in *C. elegans* [4, 67].

**Fig. 2:**
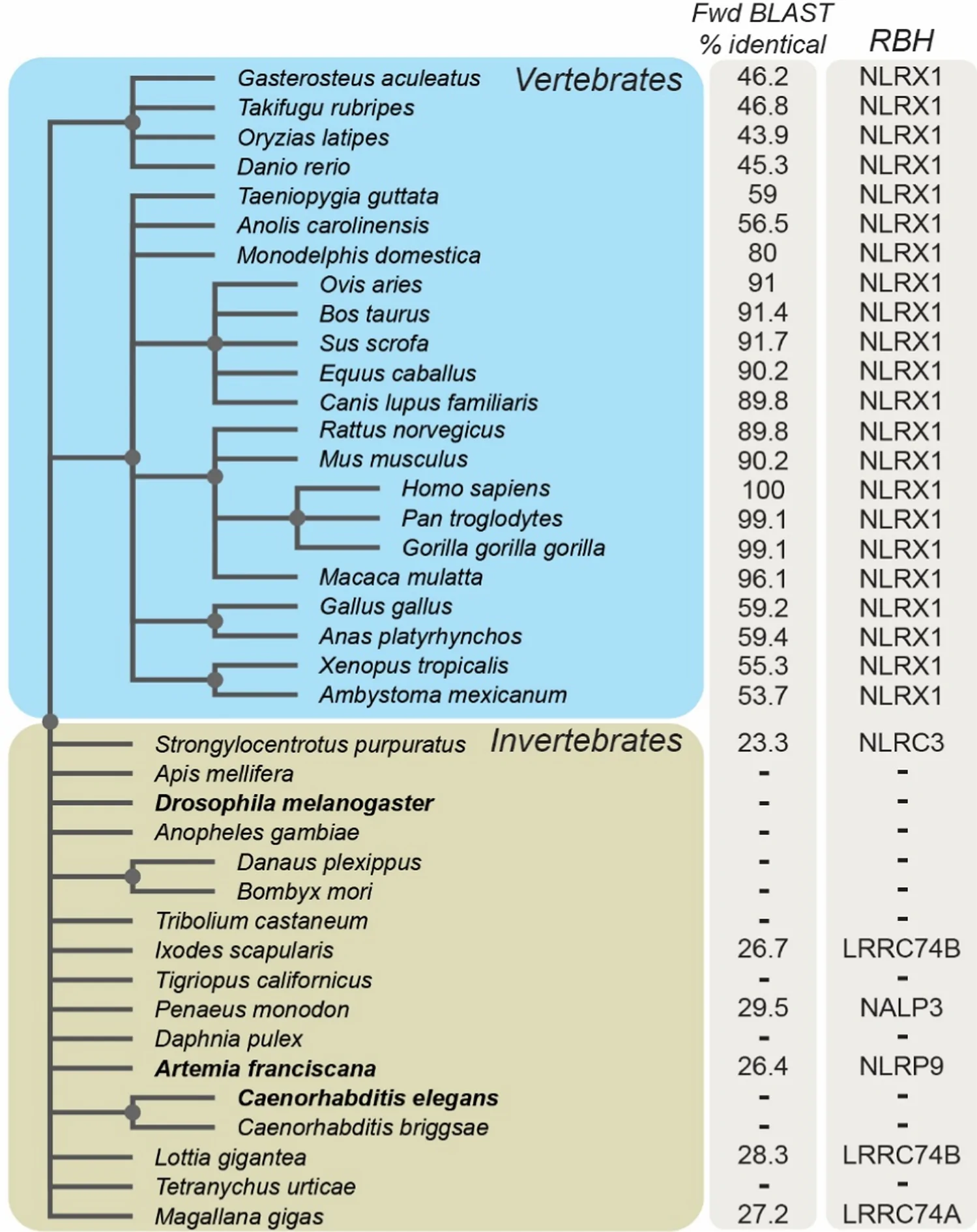
Identification of species lacking an NLRX1 ortholog. To identify potential NLRX1 orthologs across metazoans, we performed a reciprocal best hit (RBH) BLASTp analysis spanning 38 species representing major animal clades (mammals, birds, reptiles, amphibians, fishes, basal deuterostomes, crustaceans, insects, arachnids, nematodes, and molluscs). The canonical human NLRX1 protein (UniProt Q86UT6) was used as the query sequence. For each target species, a forward BLASTp search was executed against the NCBI non-redundant (nr) protein database, restricted to the target taxon, using E-value thresholds of ≤10⁻^5^ for vertebrates and ≤10⁻^3^ for invertebrates to account for expected sequence divergence. The top-scoring forward hit was then used as query in a reciprocal BLASTp against the human proteome. All searches were performed via the NCBI remote BLAST API using Biopython. The Fwd BLAST % identical column represents the percentage of similarity between human NRLX1 and the top forward BLAST hit of the respective species. The RBH column represents the top hit of the reciprocal BLAST back to the human proteome

To investigate whether absence of mPTP activity correlates with absence of NLRX1, we performed a bioinformatics search for sequence-based orthologs of the human NLRX1 protein across metazoans. Starting with the human NLRX1 sequence, candidate orthologs were identified using BLASTp. Candidates were then validated by performing a reciprocal best hit (RBH) BLASTp analysis back to the human proteome. Candidates were only considered true NLRX1 sequence orthologs if the RBH analysis did in fact return human NLRX1. This approach revealed that NLRX1 is broadly missing in all invertebrate species investigated, including *A. franciscana*, *D. melanogaster*, and *C. elegans* (Fig. 2).

A similar conclusion for *A. franciscana* was independently reached by Dr. E. Jo and Prof. H. Park (personal communication) during analysis of the recently characterized *A. franciscana* genome [16]. Moreover, search results from the ortholog database OrthoDB [34] confirm that NLRX1 orthologs solely exist in the vertebrate lineage.

These observations are consistent with evolutionary analyses of NOD-like receptor proteins [28]. Taken together, this comparative protein homology and evolutionary analysis, combined with documented presence or absence of mPTP activity, provides support that NLRX1 is required for mPTP formation.

## NLRX1, mPTP, and protection against I/R injury: a questionable quest

Recent work in NLRX1-deficient animals revealed that prevention of mPTP opening does not uniformly protect the heart. Rather, genetic ablation of NLRX1—which effectively prevents mPTP opening—was associated with increased cardiac injury following prolonged ischemia (35 min), while no detrimental or protective effect was observed after shorter ischemic episodes (20 min) [62, 63]. Thus, the presence of NLRX1 protects against cardiac I/R injury, which protection could be mediated through mPTP regulation, as was also supported by the findings that the genotype effects on I/R injury were dissipated in the presence of CsA, but also through other mechanism such as RISK pathway activation and/or reducing mitochondrial activity. Intriguingly, but speculative, Valinsky and Clapham [60] have suggested that the NLRX1-regulated mPTP was developed evolutionary in organisms that are sensitive to short-term anoxia, with mPTP attenuating tissue damage, possibly through release of mitochondrial calcium. Species where NLRX1 was evolutionary not present had used severe downregulation of metabolism as a protective mechanism against anoxic periods, a none-viable mechanisms for higher evolved organism [60].

Together, these findings challenge the concept of indiscriminate mPTP inhibition as a universal cardioprotective strategy and instead suggest that mPTP opening may exert context-dependent effects that vary with ischemic duration, metabolic state, and the dominant mechanisms of cell death engaged during reperfusion. One possible explanation for the ambiguous role of mPTP inhibition in cardiac I/R is the trade-off between beneficial (allowing mitochondrial calcium release) and detrimental effects (pore opening and loss of mitochondrial ATP synthesis). NLRX1 deletion can also increase infarct size through reducing the phosphorylation status of RISK pathway signaling molecules at reperfusion. Indeed, when the RISK pathway was activated through treatment with urocortin, NLRX1 deletion effects on RISK pathway phosphorylation status and infarct size were dissipated [62]. Of note, because of the many pleiotropic effects of NLRX1 ablation it is possible that the increased infarct size with NLRX1 ablation is not directly caused by the prevention of mPTP opening, but by some of these pleiotropic effects. However, inhibition of the pore through other means such as CsA did abrogate effects of NLRX1 ablation on infarct size, arguing that it is the effect on pore opening explaining NLRX1 effects on infarct size. Obviously, dedicated future studies are still needed to resolve these remaining uncertainties.

## Remaining questions

Although the involvement of NLRX1 in mPTP regulation was shown for CRC, mito calcium release, and CsA sensitivity assays, information from mitochondrial swelling assays or actual flux measurements through the mitochondrial pore are still awaited. It should also be emphasized that the molecular and structural mechanisms by which NLRX1 promotes pore opening remain unresolved. One possibility, proposed by Clapham and colleagues [60], is that NLRX1 impairs electron flow through the electron transport chain, thereby increasing electron leakage and mitochondrial reactive oxygen species (ROS) production. Elevated ROS levels could subsequently induce lipid peroxidation and the formation of peroxidized lipid pores (Fig. 1). In this model, specific pore-forming proteins within the mitochondrial matrix would not be required, a notion that is consistent with the absence of such proteins among the candidates identified in CRISPR screens aimed at uncovering essential components of the mitochondrial permeability transition pore (mPTP) [60]. However, it cannot be excluded at this moment that the pore is formed by NLRX1 itself (see Fig. 1). Thus, the precise molecular mechanism by which NLRX1 facilitates mPTP opening remains to be elucidated, underscoring the need for focused mechanistic studies in this area.

In conclusion, we posit that the NOD-like receptor X1 is the only known mitochondrial protein that is mandatory for the mPTP to occur. The mPTP is not a bona fide target to reduce I/R injury in all I/R conditions, probably because the (patho)physiological consequences of pore opening are a trade-off between detrimental (cell death) and beneficial (mitochondrial calcium release, PI3/Akt signaling) effects. The NLRX1 does not constitute structurally to the pore in the inner membrane of the mitochondria, but is better viewed as an essential mediator or activator of pore opening.
